# Surface Properties and Photocatalytic Activities of the Colloidal ZnS:Mn Nanocrystals Prepared at Various pH Conditions

**DOI:** 10.3390/nano5041955

**Published:** 2015-11-11

**Authors:** Jungho Heo, Cheong-Soo Hwang

**Affiliations:** Department of Chemistry, Institute of Nanosensor and Biotechnology, Center for Photofunctional Energy Materials (GRRC), Dankook University, 152 Yongin-si, Suji-ku, Jukjeon-ro, Gyunggi-do 448-701, South Korea; E-Mail: cshwang86@hotmail.com

**Keywords:** ZnS:Mn, water-dispersible, MAA capping, photocatalytic activities, methylene blue degradation, aggregations

## Abstract

Water-dispersible ZnS:Mn nanocrystals (NC) were synthesized by capping the surface with mercaptoacetic acid (MAA) molecules at three different pH conditions. The obtained ZnS:Mn-MAA NC products were physically and optically characterized by corresponding spectroscopic methods. The UV-Visible absorption spectra and PL emission spectra showed broad peaks at 310 and 590 nm, respectively. The average particle sizes measured from the HR-TEM images were 5 nm, which were also supported by the Debye-Scherrer calculations using the X-ray diffraction (XRD) data. Moreover, the surface charges and the degrees of aggregation of the ZnS:Mn-MAA NCs were determined by electrophoretic and hydrodynamic light scattering methods, indicating formation of agglomerates in water with various sizes (50–440 nm) and different surface charge values accordingly the preparation conditions of the NCs (−7.59 to −24.98 mV). Finally, the relative photocatalytic activities of the ZnS:Mn-MAA NCs were evaluated by measuring the degradation rate of methylene blue (MB) molecule in a pseudo first-order reaction condition under the UV-visible light irradiation. As a result, the ZnS:Mn-MAA NC prepared at the pH 7 showed the best photo-degradation efficiency of the MB molecule with the first-order rate constant (*k_obs_*) of 2.0 × 10^−3^·min^−1^.

## 1. Introduction

Water-dispersible semiconductor nanocrystals were developed for various important applications such as luminescent labeling agents in biological systems [1,2,3], photocatalysts for the hydrogen gas production by water splitting [4,5,6], and environmental purification agents to remove pollutants from wastewater by photodegradation reactions [7,8,9]. In the syntheses of the water-dispersible nanocrystals, several organic molecules such as sulfodiisooctyl succinate [10], mercaptoethanol [11,12], and conventional aminoacids [13,14] were found as effective surfactants to solubilize the originally hydrophobic semiconductor nanocrystals in water. However, the complicated surface properties of the nanocrystals dispersed in water have not been investigated in detail. In fact, it is generally expected that nanosized semiconductor materials would exhibit much higher catalytic efficiency than their bulk counterparts due to the increased surface area-to-volume ratio [15]. However, one should also consider that there are much more complicated factors in the water-dispersed nanocrystals such as interactions between the surface capping ligands and secondary coordination by other metal ions provided during the preparation process of the nanocrystals (NCs), which majorly results in decrease of luminescence efficiency and photocatalytic activity of the NCs [16]. Therefore, it is necessary to investigate specific surface properties such as coordination modes of the capping ligands, surface charges, and degree of aggregation in water to correctly understand the observed photocatalytic activities of the NC.

In this study, water-dispersible ZnS:Mn nanocrystals were prepared by capping their surface with polar mercaptoacetic acid (MAA) molecules at three different pH conditions (pH 2, 7, and 12). The pH conditions were selected based on the known pKa values and isoelectric point of the capping ligand (pKa_1_ = 3.64, pKa_2_ = 10.61, and pI = pH 7.13 for the MAA in water respectively) (CAS 68-11-1) at which different surface properties were expected to be observed for the ZnS:Mn nanocrystals. In particular, MAA was selected as the surface capping ligand, because it is a simple structured polar molecule, and has been known as an excellent stabilizer for other semiconductor nanocrystals such as CdX (X = S, Se and Te) [17] and ZnS [18] with a high solubility in water. Moreover, MAA does not have any aromatic functional group or alkyl side chain, which can further induce unexpected secondary interactions between the capping molecules on the nanocrystal surface, thus complicating the characterizations. To investigate the pH dependent nature of the ZnS:Mn-MAA nanocrystals in water, the corresponding surface charges of the nanocrystals were determined by the electrophoretic light scattering method [19]. Moreover, the degrees of aggregation of the nanocrystals in water were measured by the hydrodynamic light scattering method [20]. Finally, the relative potocatalytic activities of the ZnS:Mn-MAA nanocrystals were evaluated by measuring the degradation rate of an organic dye (methylene blue, MB) molecule under the UV light irradiation [21]. It has been known that pH is one of the most important factors affecting to the photocatalytic efficiency of traditional semiconductor nanocrystals [22]. However, especially for the ZnS:Mn-MAA NC, none of the studies have reported on the related surface properties such as surface charges and formation of aggregation together according to the different pH environments. Therefore, the main purpose of our study was to relate the pH dependent nature of the surface modified ZnS:Mn NCs and their observed relative photocatalytic activities in different environments.

## 2. Results and Discussion

### 2.1. Characterizations of the ZnS:Mn-MAA NC Products Prepared at Different pH Conditions

**Figure 1:**
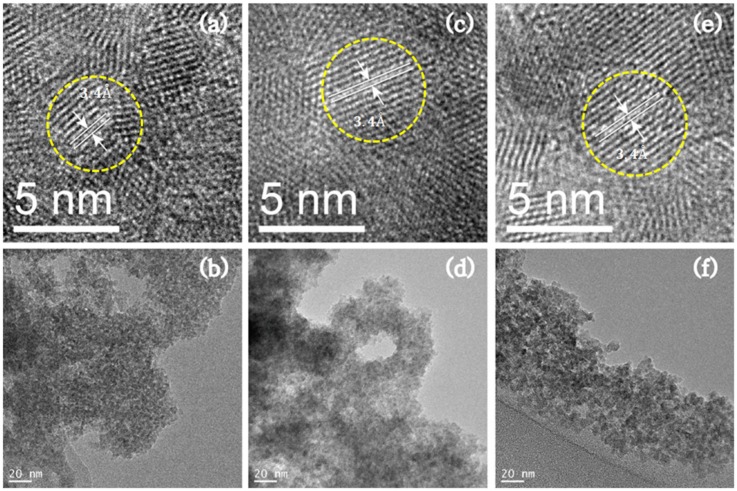
High-resolution transmission electron microscopy (HR-TEM) images of the ZnS:Mn-MAA nanocrystals (NCs) prepared at: (**a**,**b**) pH 2; (**c**,**d**) pH 7; and (**e**,**f**) pH 12.

The average particle sizes of the ZnS:Mn-MAA NCs prepared at three different pH conditions were measured from the HR-TEM images presented in Figure 1. Even though the images did not clearly show discrete individual particles, we enlarged those images as much as we could and measured about 30 identifiable particles (fringe images) to obtain the average particle sizes for the ZnS:Mn-MAA NCs. In the images, the shapes of the most particles are fairly close to a sphere, and the average of the measured particle sizes were: 3.33 nm (pH 2), 5.17 nm (pH 7), and 6.05 nm (pH 12). In the figure, little agglomerations between the particles were observed due to the evaporation of the water and alcohol mixture solvents during the sample preparation. However, the appearance of distinct lattice planes in the fringe images with about 3.4 Å lattice spacing indicated that the obtained solids are made of single crystals rather than poly-crystalline aggregates.

To determine the elemental composition of the prepared ZnS:Mn-MAA NCs and the doping concentration of the manganese (II) ions precisely, Inductively Coupled Plasma-Atomic Emission Spectrometry (ICP-AES) analyses were performed. Three trials of the sample measurements revealed that the average elemental proportions of the Mn^2+^ ions relative to the ZnS parent crystal were 1.06% (pH 2), 1.22% (pH 7), and 1.11% (pH 12). The doping concentration of the manganese (II) ion in the ZnS parent crystal was originally aimed to be 1%–2%, which had been reported as the optimum for photoluminescence (PL) efficiency for other known ZnS:Mn nanocrystals [23].

**Figure 2:**
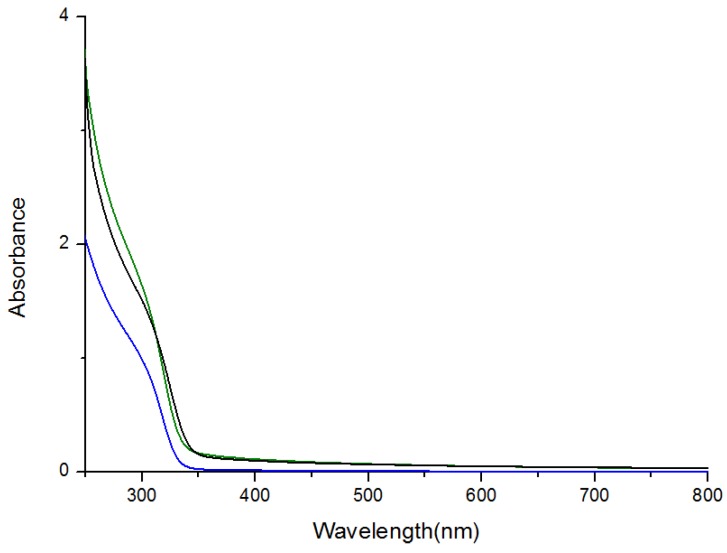
Ultra violet (UV)-visible absorption spectra of the ZnS:Mn-MAA NCs prepared at pH 2 (blue); pH 7 (green); and pH 12 (black).

**Figure 3:**
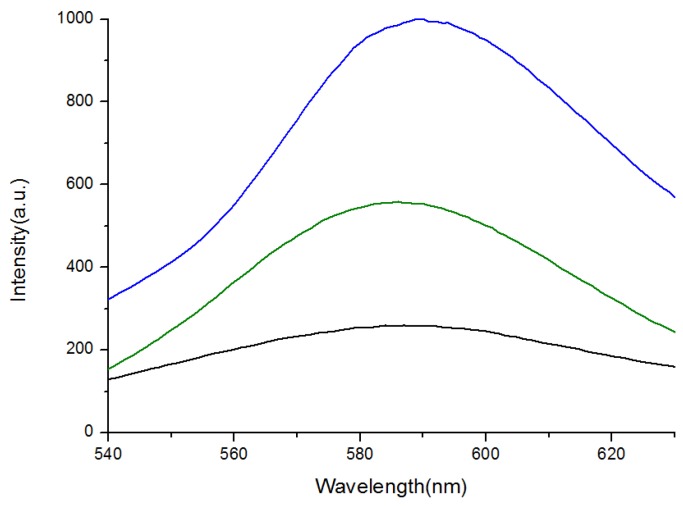
Room temperature solution photoluminescence (PL) emission spectra of the ZnS:Mn-MAA NCs prepared at pH 2 (blue); pH 7 (green); and pH 12 (black).

The optical properties of the ZnS:Mn-MAA NCs were investigated by using UV-visible and photoluminescence (PL) spectroscopy, as presented in Figure 2 and Figure 3. The PL spectra showed broad emission peaks appeared at 590 nm (pH 2), 586 nm (pH 7), and 587 nm (pH 12) that were almost identical to each other. The emission spectra were obtained by fixing the excitation wavelengths at the corresponding UV-Visible absorption peak of the NC, which were 307 nm (pH 2), 310 nm (pH 7), and 312 nm (pH 12). The dominant absorption shown in the spectrum probably resulted from the fundamental band-to-band absorption in the ZnS host [24], and the increased band gap of the ZnS:Mn nanocrystal (3.87 eV), compared to that for bulk ZnS:Mn solid (3.54 eV), is due to the well-known quantum confinement effect [25]. The yellow-orange light emissions at 590 nm were attributed to the ^4^T_1_–^6^A_1_ transitions of the dopant Mn^2+^ ions [26]. In the luminescence pathway, if the surface defect states are located close to the conduction band, the direct energy transfer from the ZnS host to the Mn^2+^ activator is significantly interrupted, which can cause weakening in the emission as well as enlarging of the Stokes shift [27]. In Figure 3, the lower PL intensities for the ZnS:Mn-MAA (pH 7 and 12) NCs were probably due to the presence of sodium ions provided by the addition of NaOH to adjust the pH condition in the preparation, since a similar phenomenon has been reported that addition of sodium salts into a thiolate ligand coated CdSe/ZnS quantum dot containing colloidal solution caused a significant luminescence quenching [28]. The PL efficiencies for ZnS:Mn-MAA NCs were measured and calculated following the method originally reported by Williams *et al*. [29,30]. This method includes calculating a relative quantum yield by comparing it to that of a standard organic dye, a 0.1 M solution of quinine sulfate in H_2_SO_4_ (purchased from Fluka) in this case [31], of which the emission wavelength and absolute quantum efficiency are known as 550 nm and 0.546, respectively (22 °C). The excitation wavelength used for the standard was fixed at the same with the ZnS:Mn-MAA NC, which was obtained from the UV-Visible absorption spectrum of the ZnS:Mn-MAA NC. As a result, the calculated relative PL efficiencies were 4.67% (pH 2), 4.02% (pH 7), and 3.98% (pH 12).

**Figure 4:**
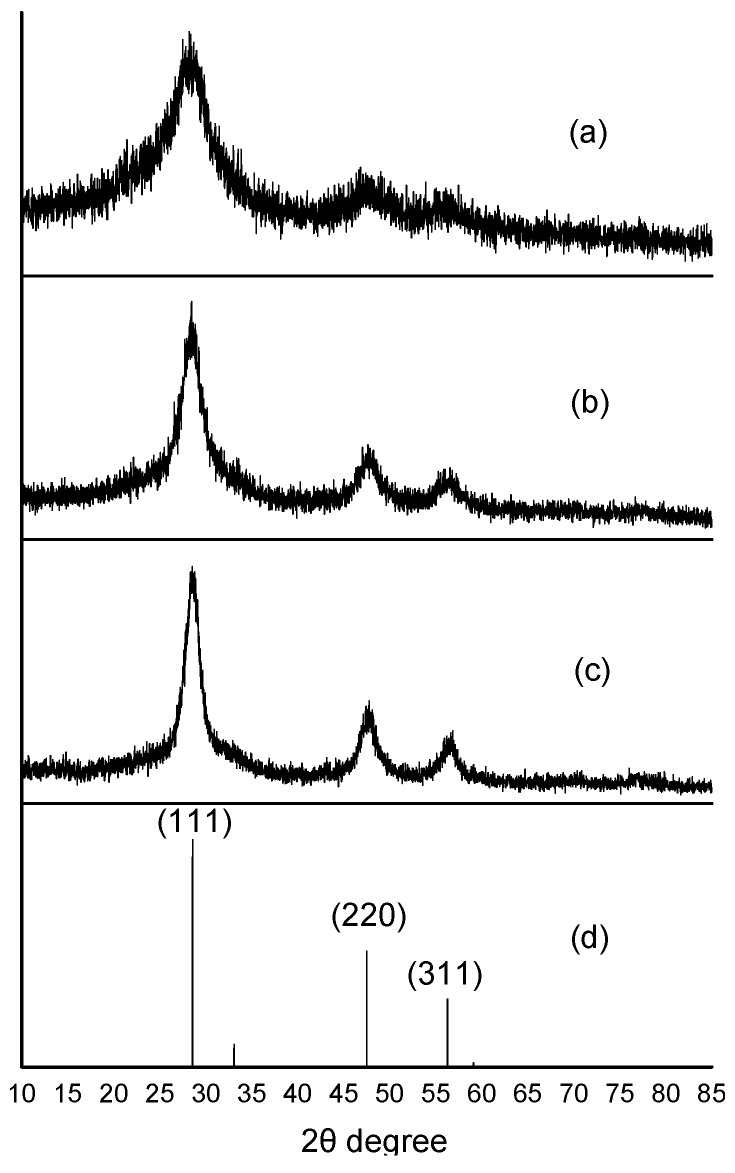
X-ray diffraction (XRD) pattern diagrams of the ZnS:Mn-MAA NCs prepared at: (**a**) pH 2; (**b**) pH 7; and (**c**) pH 12. The diagram (**d**) is a reference ZnS bulk solid pattern in a cubic zinc blend phase (JCPDS 05-0566).

In Figure 4, the wide-angle X-ray diffraction (XRD) pattern diagrams obtained from the ZnS:Mn-MAA NCs prepared at different pH conditions are presented. Although most of the peaks are broad, there were clearly identifiable (111), (220), and (311) peaks in the diagram, indicating that the ZnS:Mn-MAA NCs are in the cubic zinc blende phase (JCPDS 05-0566) [32]. In addition, we also calculated the particle sizes of the ZnS:Mn-MAA NCs by applying the Debye-Scherrer equation using the XRD peak data to compare with the particle sizes measured from the HR-TEM images [33,34]. From the measured full width at half maxima (FWHM) of the selected XRD peaks, we obtained the average particle sizes for ZnS:Mn-MAA NC as 3.51 nm (pH 2), 5.00 nm (pH 7), and 5.70 nm (pH 12), which showed very good agreements to that measured from the HR-TEM images.

**Figure 5:**
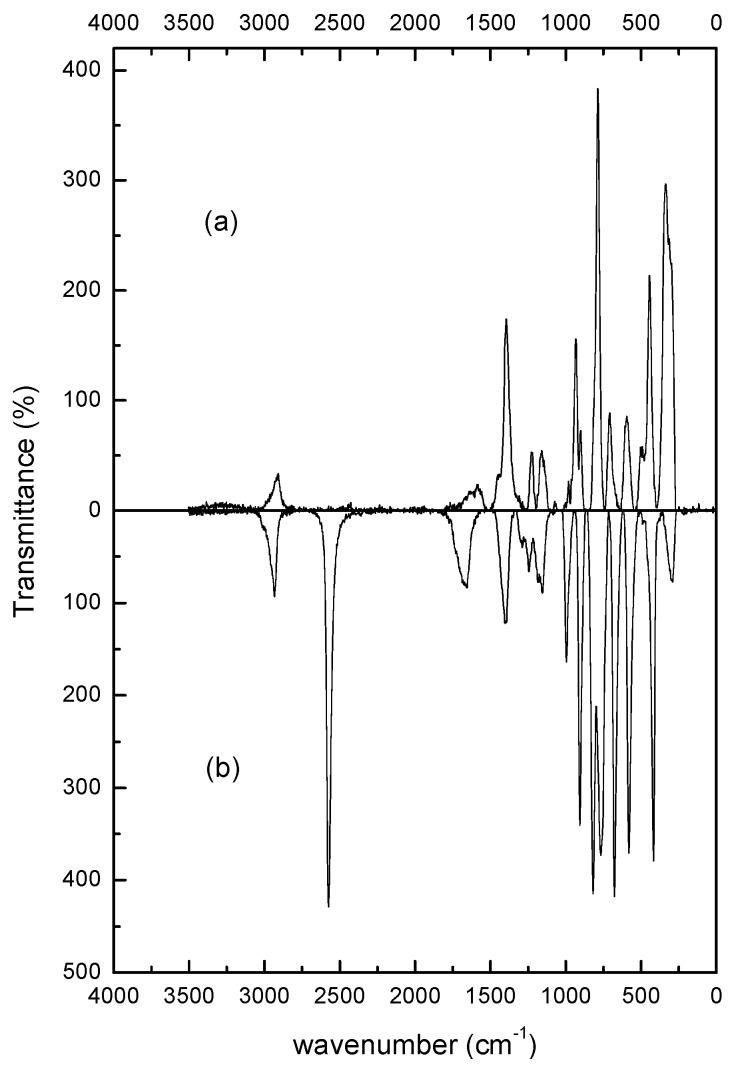
Fourier transform (FT) -Raman spectra of the: (**a**) ZnS:Mn-MAA NC (pH 12); and (**b**) Neat MAA molecule.

The MAA molecules attached on the surfaces of the ZnS:Mn-MAA NC were characterized by FT-Raman spectroscopy [35]. Figure 5 presents the FT-Raman spectra of ZnS:Mn-MAA NC prepared at pH 12 condition with that of free MAA molecules for comparison. The obtained peak data are listed in Table 1 and their assignments are also provided. The assignments of the specific vibrational modes were provided by comparing to previously reported paper regarding assignments of the FT-Raman spectrum of the ZnS:Mn-MAA associated with a computational calculation (DFT) method [36]. In the spectrum, the overall peaks from the capping MAA molecules were slightly shifted comparing to that of the neat MAA molecule since they were attached to much heavier transition metal ions in the nanocrystal lattices, and were immobilized to restrict some of vibrational modes [37,38]. The peaks appeared at 2575 cm^−1^ (S-H) for the free MAA molecules did not appear for the ZnS:Mn-MAA NC, since the hydrogen was removed by the addition of strong base at this pH condition. To remove any uncoordinated or unreacted MAA molecules, the centrifuged white solids were rapidly washed several times with cold alcohol/water solutions. As a result, the peaks that resulted from any free MAA molecules could be removed from the presented FT-Raman data for the ZnS:Mn-MAA NC. Finally, most peaks appeared in the lower region, from 400 to 1000 cm^−1^ for the ZnS:Mn-MAA NC, can be assigned as mixed banding modes of the C–H, C–C, and C–O moieties of the nanocrystal coordinated MAA molecule. The peak appearing at 335 cm^−1^ was especially assigned as a longitudinal optical phonon of the ZnS lattice [39].

**Table 1:** FT-Raman data and assignments of ZnS:Mn-MAA (mercaptoacetic acid) NC (nanocrystals) prepared at pH 12 (unit in cm^−1^).

ZnS:Mn-MAA (pH 12)	Neat MAA	Assignments
335		Zn-S phonon
444	419	δ(CCO)/ρ(OCO)
595	581	γ(CCO)/ω(OCO)
708		δ(OCO)
787	776	ν(CS)
	820	ν(CC)
934	909	ρ (CH_2_)
1395	1400	ν(OCO) + δ(CH_2_)
1583	1692	ν(C=O)
	2575	ν(SH)
2907	2932	ν(CH_2_)

ν: stretching; ω: wagging; δ: in-plane deformation; ρ: rocking.

### 2.2. Surface Properties of the ZnS:Mn-MAA NCs

To investigate the pH dependent surface properties of the ZnS:Mn-MAA nanocrystals in water, the corresponding surface charges of the nanocrystals were determined by the electrophoretic light scattering method [40]. As a result, the zeta potentials of the nanocrystals at ambient temperature were obtained as: ‒24.98 mV (ZnS:Mn-MAA-pH 2), ‒31.67 mV (ZnS:Mn-MAA-pH 7), and ‒7.59 mV (ZnS:Mn-MAA-pH 12). Considering the pH condition, the negative surface charge of the ZnS:Mn-MAA-pH 2 nanocrystal can be explained that most of the protons originally belong to the MAA molecules were ionized into the water to form their conjugate base ion moieties (MAA^−^) on the nanocrystal surface to yield the negative surface charge of the nanocrystal. For the preparation of the ZnS:Mn nanocrystal, the pH 2 condition was achieved just by adding the MAA itself without any further modification. However, considering that the carboxyl end is not so strongly acidic like a halide acid in water, one can expect that not all of the protons in the carboxyl (–COOH) end of the MAA were ionized at this environment. Therefore, obtaining the higher negative surface charge for ZnS:Mn-MAA-pH 7 nanocrystal than that of ZnS:Mn-MAA-pH 2 nanocrystal is also relevant. The addition of sodium hydroxide (NaOH) to adjust the pH 7 condition induced additional deprotonation at the carboxyl end of the MAA to produce more negatively charged nanocrystal surface even though the pH 7 is very close to the isoelectric point of the free MAA molecule in water. In this manner, obtaining much higher negative surface charge for the ZnS:Mn-MAA-pH 12 nanocrystal was expected because the SH and COOH groups of the MAA should be completely deprotonated at this pH condition. However, much lower negative surface charge value of ‒7.59 mV was actually obtained. In fact, this result was explained, when an additional elemental analysis was performed for the ZnS:Mn-MAA-pH 12 nanocrystal by the ICP-AES method. It was found that 10 atom% sodium ions compared to the ZnS contents existed in the ZnS:Mn-MAA-pH 12 nanocrystal, which were probably produced by the added NaOH to adjust the pH 12 condition in the preparation. The originally produced highly negative charge of the nanocrystal surface was probably compensated by the positive charge of the surface attached Na^+^ ions. Even though NaOH was also added for the ZnS:Mn-MAA-pH 7 nanocrystal, the elemental analysis revealed that less than 1 atom% of the sodium ions compared to the ZnS were found. Therefore, the amount of the attached sodium ions was not sufficient enough to cancel out the negative charge of the ZnS:Mn-MAA-pH 7 nanocrystal in this case.

**Figure 6:**
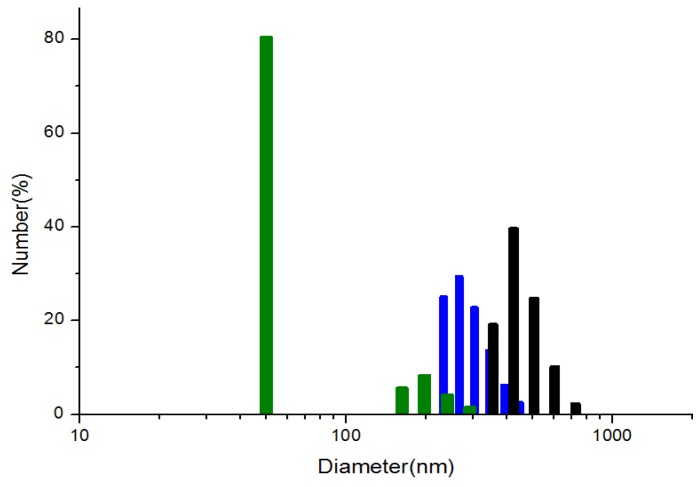
Particle size distribution diagrams of the ZnS:Mn-MAA NCs prepared at pH 2 (blue), pH 7 (green), and pH 12 (black).

Second, to investigate the pH dependent surface interactions between the water-dispersed ZnS:Mn NCs, the degree of aggregation of each nanocrystal in water was measured by the hydrodynamic light scattering method [41]. Figure 6 shows the formation of various sizes of aggregates: ZnS:Mn-MAA-pH 2 (287 nm), ZnS:Mn-MAA-pH 7 (50 nm), and ZnS:Mn-MAA-pH 12 (440 nm) nanocrystals in water. These agglomerates probably formed by the intermolecular interaction between the capping molecules on the neighboring ZnS:Mn nanocrystals, because these nanocrystals were originally found as 5 nm sized particles in the solid state according to the HR-TEM images and the Debye-Scherrer’s XRD calculations. Previously, we reported very similar intermolecular attractions between the surface capping molecules on the nanocrystal surfaces for l-glycine and l-valine capped ZnS:Mn nanocrystals [42], in which the intermolecular interaction (majorly hydrogen bonding) between the aminoacid molecules caused the formation of huge aggregates of the ZnS:Mn-aminoacid nanocrystals (from 250 nm to few micrometer sizes) in water. Compared to other water-dispersed nanocrystals, the degree of aggregation for the ZnS:Mn-MAA nanocrystals was relatively low in water, probably because of the electrostatic repulsion between the negatively charged nanocrystal surfaces [43]. The ZnS:Mn-MAA-pH 7 nanocrystal, which showed the highest negative surface charge, formed the smallest agglomerates in water, whereas the ZnS:Mn-MAA-pH 12 nanocrystal produced the largest aggregates. Moreover, the surface coordinated sodium ions probably caused the formation of sodium ion bridged complexes to form [COO–Na–OOC] moieties in water. In fact, similar phenomena have been reported previously; the addition of sodium metal ions to the 11-mercaptoundecanoic acid capped gold nanoparticles caused the formation of the metal bridged aggregates of few micrometer sizes in water [44].

### 2.3. Photocatalytic Activities of the ZnS:Mn-MAA NCs

As an application, the relative photocatalytic activities of the ZnS:Mn-MAA nanocrystals were evaluated by measuring the degradation rate of an organic dye (methylene blue, MB) molecule under the UV light irradiation [45,46]. Photocatalysis is a process by which a semiconductor material absorbs energy greater than or equal to its band gap, causing excitation of the valence band (VB) electrons into the conduction band (CB). Such charge separation forms electron hole pairs (h^+^/e^−^), generating free radicals in the system for redox of the organic substrate. The resulting free radicals such as hydroxyl (HO**^•^**) are very efficient oxidizers of organic dye molecules such as MB [47]. The detailed photo-degradation mechanism of an MB molecule in the presence of a semiconductor nanoparticle catalyst is well known in the literature [48]. A brief description of the photo-degradation mechanism of MB molecules in the presence of the ZnS:Mn nanocrystal is as follows:

ZnS:Mn + *hν* → h^+^ (VB) + e^−^ (CB) (1)

OH_2_ + h^+^ → HO^•^ + H^+^ (VB) and O_2_ + e^−^ → O_2_^−^ (CB) (2)

MB + HO^•^ → degradation products (3)

**Figure 7:**
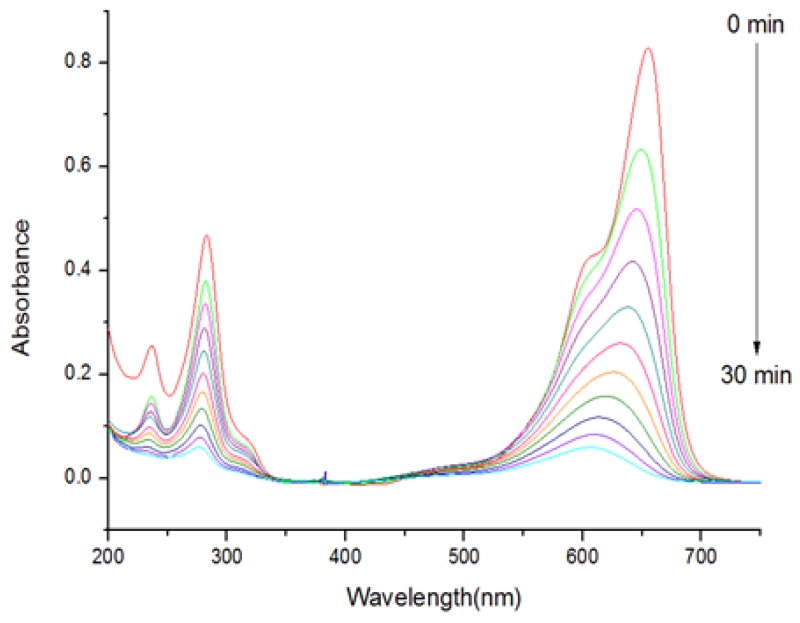
Temporal UV visible absorption spectral changes of methylene blue (MB) in the presence of ZnS:Mn-MAA-pH 7 NC under the UV light irradiation.

**Figure 8:**
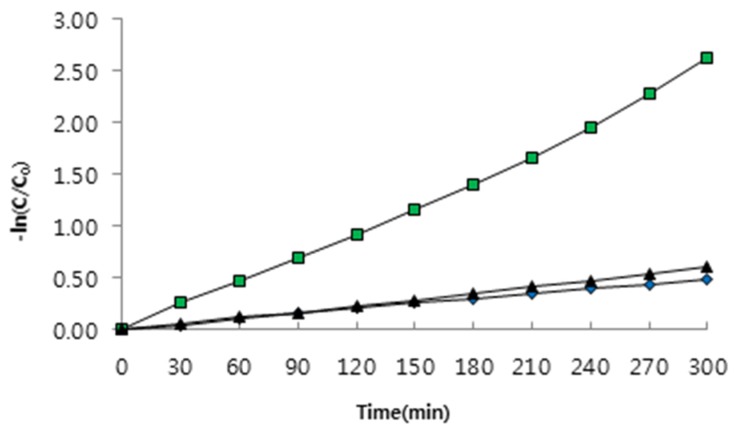
First-order kinetic plots of photo-degradation of MB in the presence of ZnS:Mn-MAA-pH 2 (blue), ZnS:Mn-MAA-pH 7 (green), and ZnS:Mn-MAA-pH 12 (black) NCs under the UV light irradiation.

In this study, relative photo-degradation efficiencies of the three surface modified ZnS:Mn nanocrystals were evaluated, as shown in Figure 7 and Figure 8. These reaction rates were measured in the pseudo first-order conditions by controlling the concentration of the reactants. The pseudo first-order rate constants (*k_obs_*) for the photo-degradation reaction of MB were calculated using the plots of ln(*C/Co*) *versus* irradiation time (*t*), in which *C* and *Co* are the concentrations of MB at a certain time and the initial state respectively, and were converted from the temporal absorbance changes in the UV-visible absorption spectra. The total reaction running times were set at 300 min for all the ZnS:Mn-MAA nanocrystals, because the absorbance of the MB molecule did not show any further change after that time period. As a result, the pseudo first-order reaction constants (*k_obs_*) calculated from the slopes in the fitting diagrams are as follows: 1.6 × 10^−3^ min^−1^ (ZnS:Mn-MAA-pH 2), 8.5 × 10^−3^ min^−1^ (ZnS:Mn-MAA-pH 7), and 2.0 × 10^−3^ min^−1^ (ZnS:Mn-MAA-pH 12). In the presented diagrams, all the calculated *R*^2^ values were greater than 0.994, indicating that the data fitted well into the straight lines. Usually the pH is known as one of the most important parameters affecting the photo-oxidation process of water [49], and in general, negatively charged photo-catalysts can attract polar water molecules more efficiently to produce hydroxyl radicals than those of the neutral ones [50]. Among these nanocrystals, the ZnS:Mn-MAA-pH 7 nanocrystal, with the highest surface charge of −31.67 mV and the largest surface area (owing to the least aggregation), showed the best photo-degradation efficiency (92% after 300 min). Moreover, the photo-degradation efficiency of the ZnS:Mn-MAA-pH 7 nanocrystal was compared to a well-known commercial reference photo-catalyst, a bulk TiO_2_ powder (Degussa, P-25) [51], and the calculated relative photo-degradation efficiency was 30% compared to the internal reference. Even though the ZnS:Mn-MAA NC showed lower photocatalytic efficiency than the commercial TiO_2_ bulk powder, ZnS:Mn nanocrystal is still interesting as photocatalysts in a system using the sun light since the ZnS:Mn can absorb visible lights with high optical transition efficiency by tuning its band gap with a size controlling method, while TiO_2_ absorbs only near UV lights which utilizes only 3%–4% of the sun light [52,53]. Moreover, ZnS:Mn nanocrystals are fairly easy to synthesize in water with a high product yield, while the preparation of TiO_2_ nanocrystal in water is quite complicated and often suffers with very low product yield [54,55]. Finally, it is notable to mention that the ZnS:Mn-MAA-pH 7 NC showed much higher photocatalytic efficiency (about 10 to 20 times) than bare ZnS [56], other ligand (mercaptoetnanol) capped ZnS:Mn NC [57], and other metals doped ZnS NC such as ZnS:Fe [58] and ZnS:Cu [59] under exactly the same pH and concentration, making the ZnS:Mn-MAA NC very useful among the ZnS based photo-catalysts.

## 3. Experimental Section

### 3.1. Instrumentations

The HR-TEM images provided in this article were taken by a JEOL JEM 1210 electron microscope (JEOL, Tokyo, Japan) with a MAG mode of 1000 to 800,000 in which the accelerating voltage was 40–120 kV. For the sample preparation, dried ZnS:Mn-MAA NC powder was dispersed in methanol and placed on a carbon-coated copper grid (300 Mesh) followed by drying under vacuum for 20 h. UV-Visible absorption spectra were recorded using a Perkin Elmer Lamda 25 spectrophotometer (Waltham, MA, USA) equipped with a deuterium/tungsten lamp and the solution photoluminescence (PL) spectra were obtained by a Perkin Elmer LS-45 spectrophotometer (Waltham, MA, USA) equipped with a 500 W Xenon lamp, 0.275 m triple grating monochrometer, and PHV 400 photomultiplier tube. The powder XRD pattern diagrams were obtained using Rigaku 300 X-ray diffractometer (Rigaku, Tokyo, Japan) with Cu Kα (1.54 Å) wavelength light source. ICP-AES elemental analyses were performed by using an Optima-430 (Perkin Elmer, Waltham, MA, USA) spectrometer equipped with an Echelle optics system and segmented array charge coupled device (SCD) detector. For the surface characterization of the ZnS:Mn-MAA NC, FT-Raman spectrum was recorded by a Bruker FRA106/s spectrophotometer with a resolution of 1 cm^−1^. Finally, the surface charges and the degrees of aggregations of the ZnS:Mn-MAA NCs in water were measured by using an ELS-8000 Zeta-PSA analyzer (Otsuka Electronics, Tokyo, Japan) equipped with a 10 mW He/Ne laser light source (KBSI).

### 3.2. Syntheses of the ZnS:Mn-MAA NCs

ZnS:Mn-MAA nanocrystals were synthesized following a slight modification of the previously reported aqueous synthesis of MAA capped ZnS:Mn NCs via the formation of zinc (II) ion containing reactive intermediate complexes [36]. The additional modifications were performed to adjust different pH conditions during the preparations. A 50 mL aqueous solution of ZnSO_4_·5H_2_O (1.44 g, 5 mmol) was slowly added to a 50 mL aqueous solution containing 10 mmol of MAA at 5 °C (ice-water bath). The solution was warmed to ambient temperature after 1 h stirring. MnSO_4_·H_2_O (0.02 g, 0.1 mmol) and Na_2_S (0.40 g, 5 mmol) were dissolved in 20 mL DI water. The resulting mixture was subsequently transferred to the flask containing the [Zn-MAA] complexes under vigorous stirring. The subjected pH conditions were adjusted by the addition of 0.01 M NaOH aqueous solution to the mixture, measuring the pH by using a digital pH meter. The resulting solution was refluxed for 10 h. Slow cooling at ambient temperature and the addition of ethanol afforded an off-white precipitate at the bottom of the flask. Finally, the obtained solids were separated by centrifuging and decanting the supernatant. The solids were then dried for 24 h in a vacuum oven. The detailed experimental data are listed in Table 2.

**Table 2:** Experimental data summary of ZnS:Mn-MAA NCs prepared at different pH conditions.

Experimental Data	pH 2	pH 7	pH 12
UV/Vis absorption (λ_max_, nm)	307	310	312
PL emission (λ_max_, nm)	590	586	587
PL efficiencies (%)	4.67	4.02	3.98
concentration Mn dopant ICP-AES (%)	1.06	1.22	1.11
Average particle size HR-TEM (nm)	3.33	5.17	6.05
Average particle size XRD (nm)	3.51	5.00	5.70
Zeta potentials (mV)	−24.98	−31.67	−7.59
Average size of aggregates in water DLS (nm)	287	50	440

### 3.3. Photocatalytic Experiments

The photocatalytic reactions were performed using a 300 W xenon lamp (Newport) as the light source (emission range from 185 to 2000 nm), which was placed at the side of a 1.0 L capacity Pyrex-glass cell (Sigma-Aldrich, Seoul, Korea) in a dark room. The glass cell was filled with 0.5 L of aqueous mixture solution containing 100 mg of MB and 5 mg of the ZnS:Mn-MAA NC as the photo-catalyst. Under this condition, the molar concentration ratio of [ZnS:Mn]:[MB] was 1:150 according to the ICP-AES elemental analyses for the ZnS:Mn NC. The resulting solution was stirred using a magnetic bar, and a magnetic stirrer was placed under the glass cell during the reaction. The degradation of MB was monitored by aliquoting 10 mL of the mixture solution at the 30 min irradiation time intervals. Subsequently, the degradation reaction rate was calculated by measuring the change in the absorbance of the MB dye solution. The absorption spectra were recorded by measuring the absorbance at 664 nm corresponding to the maximum absorption wavelength of the MB molecule with UV-Visible spectroscopy. Analogous control experiments were performed without ZnS:Mn NC (blank). Moreover, to exclude the heat factor from the calculation, generated by the UV lamp during the degradation reaction, the same reaction was performed without UV light irradiation by just heating using a hot plate (up to ~90 °C, 300 min) placed under the glass cell, confirming that no degradation reaction occurred without UV light irradiation.

## 4. Conclusions

Recently, low dimensional semiconductor nanocrystals have received attention as novel types of photocatalytic materials for the purification of wastewater and the hydrogen production process. Since ZnS:Mn NCs can provide a novel platform to attract polar molecules such as water, they can be used for a variety of applications especially as a photocatalyst for the degradation of pollutants in wastewater or the hydrogen production by the water splitting process. In this study, MAA capped ZnS:Mn NCs were successfully synthesized at different pH conditions, and the corresponding surface properties were investigated using spectroscopic methods. In addition, the related physical and optical properties were also investigated. The as-prepared water-dispersible MAA capped ZnS:Mn NCs indeed showed remarkable physicochemical properties for further applications in advanced photocatalysis systems. However, as demonstrated in this study, both the degree of aggregation in water and the pH dependent nature of the MAA, as the capping ligand for the ZnS:Mn NCs, are very critical factors to be considered for further applications in any commercial device.
